# Nanodelivery Strategies for Skin Diseases with Barrier Impairment: Focusing on Ceramides and Glucocorticoids

**DOI:** 10.3390/nano12020275

**Published:** 2022-01-15

**Authors:** Cíntia Almeida, Patrícia Filipe, Catarina Rosado, Catarina Pereira-Leite

**Affiliations:** 1 CBIOS—Universidade Lusófona’s Research Center for Biosciences & Health Technologies, Campo Grande 376, 1749-024 Lisboa, Portugal; cintia.almeida@ulusofona.pt (C.A.); patricia.cfilipe@gmail.com (P.F.); 2 LAQV, REQUIMTE, Departamento de Ciências Químicas, Faculdade de Farmácia, Universidade do Porto, Rua de Jorge Viterbo Ferreira 228, 4050-313 Porto, Portugal

**Keywords:** stratum corneum, skin disorders, ceramides, glucocorticoids, nanoparticles, atopic dermatitis, psoriasis, xerosis, ichthyosis

## Abstract

The human epidermis has a characteristic lipidic composition in the stratum corneum, where ceramides play a crucial role in the skin barrier homeostasis and in water-holding capacity. Several skin diseases, such as atopic dermatitis and psoriasis, exhibit a dysfunction in the lipid barrier with altered ceramide levels and increased loss of transepidermal water. Glucocorticoids are normally employed in the therapeutical management of these pathologies. However, they have shown a poor safety profile and reduced treatment efficiency. The main objective of this review is to, within the framework of the limitations of the currently available therapeutical approaches, establish the relevance of nanocarriers as a safe and efficient delivery strategy for glucocorticoids and ceramides in the topical treatment of skin disorders with barrier impairment.

## 1. Introduction

Skin diseases are pathological disorders that affect the human skin compromising its functions, such as protection against toxic substances and ultraviolet (UV) light, trauma, prevention of water loss, body temperature regulation, as well as endocrine and sensory function [1]. According to Karimkhani et al. [2], skin diseases represent 1.8% of the global burden of disease measured in DALYs (disability-adjusted life years). Those that affect skin barrier function are particularly relevant. It is estimated that atopic dermatitis (AD) and psoriasis together affect 12% of adults worldwide [3,4]. AD is an inflammatory skin disease with a huge impact on patients’ daily lives and affects around 1–3% of the adult population in Europe [5]. AD-related out-of-pocket costs pose an extra spending mean amount of €927 by every patient per year [5]. In Europe, AD and psoriasis represent the skin diseases that triggered the most visits to the dermatologist [6], with the burden of psoriasis being greater in patients from European high-income countries [7]. In the European Union, the economic burden of psoriasis per patient/year varies from €5000 (Germany) to €12,000 (Sweden), while the total cost of psoriasis therapy is more than $50 billion per year in the United States of America [8]. 

In the present review the role of the epidermis will be highlighted, and the main skin diseases associated with epidermal barrier disruption—xerosis, ichthyosis, atopic dermatitis, and psoriasis—will be briefly described. However, its main objective is to, within the framework of the limitations of the currently available therapeutical approaches, establish the relevance of nanocarriers as a safe and efficient delivery strategy for glucocorticoids and ceramides in the topical treatment of these disorders. 

### 1.1. Epidermis and Skin Barrier Function

**Figure 1:**
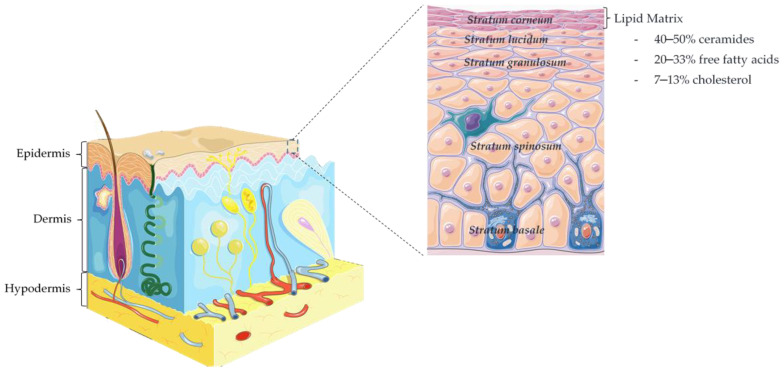
Schematic representation of skin layers and epidermis structure.

The skin structure is formed by three main layers, namely hypodermis (up to 3 cm thick), dermis (3–5 mm thick), and epidermis (50–100 µm thick) [9], as showed in Figure 1. The epidermis, the outside layer, is comprised of *stratum basale* (SB)*, stratum spinosum* (SS)*, stratum granulosum* (SG)*, stratum lucidum* (SL)*,* and *stratum corneum* (SC). The SC desquamation is balanced with the proliferation and migration from the basal layer epidermal cells, the keratinocytes. Those are the most abundant cells of the epidermis and during their course towards the outermost layer undergo transformations in both structure and composition [9,10]. The terminal differentiation occurs in the interface of the SG-SC, where the viable keratinocytes are transformed into corneocytes containing keratin and enclosed by a cross-linked protein envelope, named cornified envelope (CE), and by a lipid layer with ceramides, free fatty acids, and cholesterol [9,11]. Corneocytes are interconnected through protein complexes, the corneodesmosomes, which are important for the cohesion of the SC. During maturation of the SC, these connective links are totally degraded by proteolytic enzymes, leading to epidermal desquamation [12]. 

The SC is pivotal to the function of the skin barrier [9]. Despite being only 10–20 μm thick, in comparison with an average skin thickness of 1.2–1.3 mm [13], it is composed by highly ordered layers of corneocytes with multilamellar lipid sheets [14]. The lipid composition represents around 5–15% in relation to the other components of the SC, namely, proteins (75–80%) and unknown materials (5–10%) [9,15]. Notwithstanding being a relatively low percentage, these extracellular SC lipids play the main role in the (im)permeability of the skin, being the main skin penetration pathway of most substances the intercellular route [9,16,17]. A mixture of polar and nonpolar lipids can be found in the SC—free fatty acids (FFA; mostly saturated and long chain, between C16 and C26), ceramides (CERs), and cholesterol [10,18]. The FFA are found in higher quantities in the upper layers of the SC than in the inner layers, but this amount can vary with age, sex, anatomical site, and certain pathologies, as well as within the seasons of the year [9,19]. 

CERs are the most expressive lipid class in the human SC, representing about 50% of its lipid content, and 10 different types of free CERs were already identified [18,20,21]. CERs are polar sphingolipids composed of a fatty acid and a sphingosine base, varying their molecular base chains from 18 to 22 carbons. The production of CERs in the human SC is regulated by two key enzymes, sphingomyelinase and beta-glucocerebrosidase, whereas their degradation is regulated by ceramidase [22,23]. Their nomenclature is based on different arrangements of the fatty acid (α-hydroxy- A; non-hydroxy- N; ester-linked omega-hydroxy- EO) and sphingoid base (sphingosine- S; phytosphingosine- P; dihydrosphingosine- DS; 6-hydroxysphingosine- H), thus creating distinct classes of CERs [24]. These variations on the CERs’ chemical composition and their quantity directly influences human skin properties. The existence of long-chain CERs in the SC increases the P-ceramide levels, as well as the proportions of cutaneous proteins and lipids, contributing to an effective epidermal barrier function [25]. 

Interestingly, to evaluate the importance of CERs, in particular CER[NS] (a combination of nonhydroxy fatty acids and sphingosine base), Joo et al. [18] measured and examined these CER species and its relationship with barrier function in healthy human volunteers. SC CER[NS] species were identified and quantified in decreasing order—C24Cer, C16Cer, C18Cer, C24:1Cer, C20Cer, C18:1 ≅ C16SM. There is a wide interindividual variation in the total amount of CER[NS], corresponding with a variation coefficient of 57%. The authors demonstrated that there were statistically significant correlations of C18Cer and C20Cer with transepidermal water loss (TEWL), where shorter chain CER[NS] (C16Cer and C18Cer) had positive correlation, while longer chain (C20Cer, C24Cer and C24:1Cer) were negatively correlated. From these results, CERs with longer fatty acids and lower polarity may be beneficial for cutaneous barrier function, and the relative balance of CER species is more important than their amounts [18].

### 1.2. Skin Pathologies Associated with Barrier Impairment

The composition and organization of lipids in the SC can be affected by several factors, thus impacting the barrier function. However, these alterations normally lead to a restorative sequence of processes, causing an increase in cholesterol, FFA, and sphingolipid synthesis within a few hours [26].

External factors such as chemicals, sanitizing formulations, environmental pollutants, and some pharmaceutical ingredients, as well as skin exposure to physical insults such as elevated temperature or low ambient relative humidity, can cause higher water loss, skin dryness, and even itching [27,28]. Furthermore, exposure to UV radiation may cause the destruction of microbiota that normally colonize the skin, leading to imbalance and resulting in infectious processes, which, in turn, may exacerbate skin lesions [27].

In addition to external interferences, the composition and organization of SC lipids can be influenced by internal physiological and biological conditions. With age, there is a decrease in the levels of all major SC lipids, mainly CERs, and reports in the literature even indicate a reduction of around 10–15% per decade after 20 years of age [9,29]. Jin et al. [29] established that there was a marked increase in ceramidase activity that resulted in a higher rate of CERs degradation in the SC of aged skin, which was linked to xerosis. Emotional factors, high stress levels, and sleep deprivation were also linked to skin barrier function homeostasis, which could be related to stress-induced cytokine secretion [30], triggering off skin disorders.

**Figure 2:**
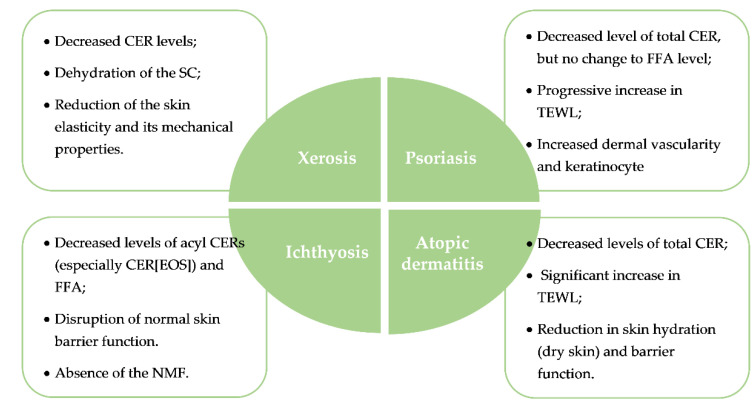
Brief schematic representation of some common skin diseases associated with epidermal barrier impairment and their main effects on barrier function of skin.

Several skin diseases were associated with epidermal barrier impairment and perturbations of the SC lipid composition [19,26] such as xerosis, ichthyosis, AD, and psoriasis [9]. The main characteristics of these pathological skin disorders are summarized in Figure 2.

#### 1.2.1. Xerosis

Xerosis or dry skin is a common condition that becomes more prevalent with age. In addition to a lower SC moisture content, it can be characterized by alterations in desquamation enzymes, a reduction in skin elasticity and mechanical properties, as well as impairment of the skin barrier function [31]. In dry skin, the proteolytic and lipid enzymes are downregulated, leading to high epidermal flaking and an increase in the skin surface area for bacterial adhesion [32,33]. Severe xerotic skin is also characterized by epidermal roughness, pruritus, and even superficial fissures, thus allowing the entry of xenobiotics into the skin [34]. It can be exacerbated in the winter, since external environment influences the composition of CERs as well as the total lipid organization of SC. Additionally, very warm environments can cause sweating, thus leading to dry and itchy skin [27]. Rawlings et al. [35] established a correlation between CER levels and xerosis degree, showing that the level in soap-induced winter xerotic skin is on average 60% lower than in normal skin [35]. The study carried out by Jin et al. [29] determined the activity of beta-glucocerebrosidase and ceramidase enzymes on the forearm skin of subjects aged up to 80 years who suffer from dry skin. Results showed that the activity of beta-glucocerebrosidase remained unchanged, while that of ceramidase was significantly lower in volunteers over 40 years old, compared to that of younger volunteers [29].

#### 1.2.2. Ichthyosis

Ichthyosis is a hereditary disorder resulting in chronic dry skin. It is characterized by a keratinization defect, the absence of the “natural moisturizing factor” (NMF) resulting in dry, thick, and generalized scaly skin [36]. The different types of ichthyosis vary in physical appearance, symptom severity, and pattern of inheritance, such as autosomal or dominant [37]. Inherited mutations and loss-of-function in the filaggrin gene (FLG) was shown to cause moderate to severe ichthyosis vulgaris [38]. Additionally, molecular biology studies have identified mutations in genes and enzyme defects that cause ichthyosis which are related to the synthesis of epidermal CERs [39,40]. A study conducted by Eckl et al. [41] showed that a mutation in the human CERs3 gene, that causes autosomal recessive congenital ichthyosis (ARCI), is linked to ceramide synthase 3 inactivity, crucial for the synthesis of CERs with very long acyl chains. Furthermore, the in vitro results indicated a cornification disorder with an inadequate barrier function [41].

#### 1.2.3. Atopic Dermatitis

Also named eczema, AD is a heterogenous chronic inflammatory disease that is thought to be triggered by an association of environmental factors in genetically susceptible individuals [42,43]. Several factors are related to the onset of AD as well as its progression: genetic susceptibility, mutations of the protein filaggrin, epidermal barrier dysfunction, immune disorders, cutaneous inflammation, dysbiosis of the skin microbiota, and a deficiency of CERs in the SC [43,44,45]. Individuals with AD usually have impaired skin barrier function and this dysfunction of the SC leads to a high susceptibility to irritant agents and allergens, and is usually manifested through redness, dryness, pruritic papules, plaques, and lichenification [46,47].

Several studies associated the onset of AD with low levels of CERs in the SC. To understand the biochemical pathway of CER deficiency in AD, Chang-Yi et al. [48] compared the expression levels of a sphingolipid activator protein, prosaposin, in the epidermis of normal and atopic dermatitis skin. The level of prosaposin in atopic dermatitis skin was decreased by 30%, in comparison to that of controls, indicating that this impairment is possibly associated with irregular SC formation in atopic skin via lower stimulation of sphingomyelinase or beta-glucocerebrosidase [48]. In 2002, Arikawa et al. [49] performed a study using a quantitative assay for sphingosine by N-acetylation, where they concluded that the SC of patients suffering from AD presented decreased levels of sphingosine, while the levels of *Staphylococcus aureus* were high, in contrast to healthy subjects [49]. More recently, Janssens et al. [50] compared the SC CERs profile between AD patients and healthy individuals and also correlated the chain length of CERs with the development of AD. They reported a reduction in the average length of the CER chain, followed by significantly elevated C34 CERs levels and decreased ω-esters of (ω)-O-acyl-CER levels [50,51]. In addition, their study showed that the changes in CER chain length were strongly correlated with abnormal lipid organization and increase in TEWL, as well with disease severity and levels of NMF derived from filaggrin [50,52].

#### 1.2.4. Psoriasis

A chronic inflammatory and immune-mediated disorder, psoriasis affects the skin and joints, causing negative effects on patient quality of life, having an estimated global prevalence of 2–3% [53]. The onset of psoriasis can be triggered by various factors, such as genetic predisposition, environmental factors, and stress. It is a disorder that involves both the innate and the adaptive immune response, in which keratinocytes, dendritic cells, and T cells have central roles [53,54]. Psoriasis is a cutaneous and systemic disorder that is characterized by progressive increase in TEWL, disrupted epidermal homeostasis, and decreased skin barrier function [55]. Recent studies also corroborated the fact that there are changes in the CERs profile of psoriasis patients. Tawada et al. [56] observed significant differences in the fatty acid composition profiles in the subclasses CER [ADS], [NP], [NH], and [AP] in the SC, in addition to a reduction in proportion of CERs with long-chain fatty acids in psoriasis patients [56]. The increase in the levels of CERs containing sphingosine and sphinganine, the increase in the expression of ceramidase, and the reduction in the presence of CERs containing phytosphingosine in their molecular structure have also been observed in the psoriatic epidermis [57,58]. These studies suggest a correlation of these changes with decreased skin hydration, with inadequate adherence between corneocytes, and with the clinical severity of psoriasis. CERs play a crucial role in several signaling pathways, stimulating apoptotic signaling molecules, such as PKC-α (protein kinase C-alpha) and JNK (c-jun N-terminal kinase) [59]. Lew and coworkers [60] observed that the levels of CERs and JNK in nonlesional epidermis were higher than in lesional epidermis and that PKC-α was only revealed in nonlesional epidermis [60].

Several approaches were attempted for the therapy of skin diseases associated with barrier impairment, but to date fully effective or safe responses for their management are still lacking. Conventional treatment includes the use of topical glucocorticoids, due to their anti-inflammatory activity. In the case of psoriasis, other strategies were used, such as topical therapy with vitamin D derivatives or salicylic acid, and systemic therapy (immunosuppressants, monoclonal antibodies), and biological drugs. These approaches are often found to be ineffective and costly [61,62,63]. Nevertheless, barrier reparation is possible and can be normalized, especially if the skin is supplied with exogenous lipids [9]. Therefore, to achieve an improvement in the treatment of these skin diseases, nanocarrier systems can be used to deliver drugs or bioactive compounds, increasing their penetration through the SC directly to the affected region and target cells [64]. The inclusion of CERs in these nanocarriers may also be beneficial to promote barrier repair. In the next section, their applicability as topical therapeutic agents for the treatment of skin disorders will be presented.

## 2. Nanocarriers for Topical Delivery

The nanoscience and nanotechnology field was triggering many advances for the diagnosis, treatment, and prevention of various diseases. Nanocarriers are defined as submicron-sized (below 1 µm) colloidal particles and may be made of different materials, including biodegradable materials such as natural or synthetic polymers and lipids [65]. They are usually not drugs themselves, but can be loaded with drugs, active substances, genes or radioactive materials, and their surface can be functionalized to be directed to a specific site of action [66,67]. Nanocarriers can be organized in two major groups: organic and inorganic nanocarriers. Organic nanocarriers include polymeric nanoparticles and lipid-based nanoparticles and are usually biocompatible, versatile, and enable the incorporation of a wide range of active compounds. However, they can present instability issues, in particular the lipid-based nanoparticles [68]. On the other hand, inorganic nanocarriers include silica nanoparticles and metallic nanoparticles, which usually can be loaded with limited amounts of drugs and may present safety issues, even though they were highly explored for diagnostic imaging [69]. In Table 1, nanocarriers were grouped according to their composition and their general advantages and disadvantages are presented, together with their main potential for topical skin applications. Despite their intrinsic disadvantages, nanocarriers may offer increased bioavailability, biocompatibility, as well as a controlled release, reducing the number of administrations and the administered dose, thus granting safety, treatment efficacy, and patient compliance [70]. Nanocarriers are also valuable to protect the drug from physicochemical and enzymatic degradation, increasing their stability without affecting the drug bioavailability, leading once again to drug accumulation at the target site and reducing toxicity [71].

The topical route offers several advantages, such as easy access and large surface area for drugs absorption, it avoids first pass hepatic metabolism, and is less invasive and painful, when compared to the subcutaneous route. Nanocarriers were considered for topical delivery of drugs, since they were enhancing skin targeting and skin permeation, enabling the development of topical therapies formerly unutilized due to low transdermal penetration [72]. One strategy to increase drugs’ skin permeation and retention time is the possibility to tailor nanoparticle properties by modifying their surface charge. Studies showed that negatively charged nanoparticles improve skin permeation, while positively charged nanoparticles favor skin retention [73].

Considering the potential of nanocarriers and topical delivery, the scientific community combined these strategies to improve the management of skin diseases associated with epidermal barrier impairment. In particular, advances in the topical nanodelivery of glucocorticoids and CERs will be presented in the next subsections.

- Silver nanoparticles (AgNPs)

### 2.1. Topical Nanodelivery of Glucocorticoids

Glucocorticoids are a class of corticosteroids that have several clinical uses in the therapy of dermal disorders [91]. Commercially, there are several topical glucocorticoid formulations available, displaying different efficacy levels [92]. They are widely used as the first-line anti-inflammatory treatment and reduce disease recurrence when correctly applied at skin lesions [43]. The anti-inflammatory effect is obtained via the inhibition of inflammatory transcription factors, such as AP-1, as well as reduction of phospholipase A2 and prostaglandins release [93].

Although the topical route is highly attractive, due to large skin surface area for absorption, the penetration of glucocorticoids through the SC was shown to be limited, leading to increased dosage frequency, which can cause local adverse effects and decreased patient adherence [94]. Moreover, topical glucocorticoids should not be applied to sensitive areas of the skin, in prolonged treatments or in high doses, due to the occurrence of adverse events, such as immunosuppression, skin irritation, or stretch marks [95]. Furthermore, when the outcomes of topical therapy are insufficient or when the extent of the disease makes the use of topical therapy unviable, it is necessary to consider systemic therapeutic strategies, which also predispose to the occurrence of adverse effects [96].

The use of the traditional topical therapies to deliver drugs is often unspecific and the skin penetration can be very low with high variation. Topical formulations containing nanocarriers may provide the means for effectively improving skin penetration and reducing the side effects associated with glucocorticoids [97]. This triggered studies on nanotechnological-based approaches to improve the safety and the efficacy of topical glucocorticoid therapy (Table 2).

#### 2.1.1. Polymeric Nanoparticles

Polymeric nanoparticles were explored for the delivery of drugs to treat various diseases and dermatological disorders. In 2012, Rosado and coworkers [100] produced poly(ε-caprolactone) nanoparticles loaded with hydrocortisone and tested the drug release kinetics and its skin permeation comparatively to free hydrocortisone for AD treatment. The in vitro tests showed a prolonged release and better skin permeation using Franz cells compared to that of the free drug, and the cytotoxicity studies did not show drug toxicity of hydrocortisone-loaded nanoparticles [100].

Hussain et al. [101] evaluated the anti-inflammatory efficacy of the transcutaneous delivery of hydrocortisone-loaded chitosan nanoparticles for the treatment of AD using the NC/Nga mouse model. The developed chitosan nanoparticles displayed positive surface charge (+40 mV), mean particle size of 214 nm, and an encapsulation efficiency of 79%. The in vivo tests showed that the nanoparticle-based formulations loading hydrocortisone were efficient in reducing the severity of the pathological features of AD, including the production and release of IgE, histamine, prostaglandin-E2, vascular endothelial growth factor-α (VEGF-α), and others AD-associated inflammatory mediators [101].

Beber and collaborators [102] developed cationic polymeric nanocapsules prepared with Eudragit^®^ RS 100 and loaded with dexamethasone, which were further incorporated in a hydrogel for the topical treatment of psoriasis. The nanocapsules presented mean particle size of 140 nm, zeta potential of +11.4 mV and encapsulation efficiency of 80%. In vitro tests showed sustained release of dexamethasone from the formulation and the nanoencapsulation increased drug retention in the epidermis of abdominal porcine skin, without passing to deeper layers of skin [102].

Pandey and coauthors [103] developed betamethasone-loaded hyaluronic acid-coated chitosan nanoparticles with 290–310 nm, positive zeta potential (ca. +60 mV), and an encapsulation efficiency of 85–90%. The chitosan nanoparticles loading betamethasone and coated with hyaluronic acid showed enhanced sustained drug release and drug retention in ex-vivo Wistar albino rats epidermis and dermis, in contrast to uncoated nanoparticles [103].

Dong et al. [99] recently developed pH-sensitive Eudragit^®^ L100 nanoparticles for the (trans)dermal delivery of dexamethasone. The drug-loaded nanoparticles displayed average size and polydispersity index (PDI) of 303 nm and 0.074 (respectively) and the drug release was triggered above pH 5.9. Ex vivo permeation studies with intact and barrier-disrupted porcine skin showed that polymeric nanoparticles enhanced the penetration of dexamethasone in comparison with a cream. Remarkably, the use of these pH-sensitive nanoparticles led to a faster drug release and further drug penetration in the case of barrier-disrupted skin, suggesting that these nanocarriers may be specially interesting to load glucocorticoids to lesional skin [99].

#### 2.1.2. Lipid-Based Nanoparticles

Lipid-based nanoparticles were also considered to ameliorate the glucocorticoid topical delivery. Bikkad et al. [106] developed halobetasol propionate-loaded SLN using a 3^2^ factorial design and the optimized batch displayed an interesting size (200 nm) and entrapment efficiency (93%) for topical application. After incorporating the drug-loaded SLN into a gel formulation, the authors performed preclinical studies on rabbits and concluded that the SLN based gel was less irritant than a marketed formulation, which may ultimately improve skin tolerability and patient adherence [106].

SLNs loaded with triamcinolone acetonide (TA) and produced through the emulsification-ultrasonication method were developed by Pradhan et al. [105]. For that, several lipids were explored and the resulting nanocarriers were characterized in terms of physicochemical features. The selected formulation was composed of Compritol^®^ 888 ATO, displaying a mean particle size of 114 nm, zeta potential of -35.8 mV, and encapsulation efficiency of 82%. The in vitro tests showed a prolonged drug release compared with the TA suspension using a dialysis membrane. Moreover, TA-loaded SLNs exhibited twice as much epidermal distribution as TA suspension [105].

NLCs were used in topical corticosteroid therapies. Pradhan et al. [107] developed fluocinolone acetonide (FA)-loaded NLCs to evaluate its potential as a topical delivery system for psoriasis treatment. The studies showed that the mean particle size of the optimized formulation was 153 nm with a PDI of 0.01 and the entrapment efficiency was found to be 92%. The studies showed a significant amount of FA deposited in the epidermal and dermal layer of skin and the authors concluded that FA-loaded NLC may be a promising nanocarrier system for psoriasis treatment [107]. Later, the same authors evaluated the effect of TA-loaded NLCs for psoriasis management. The optimized formulation displayed a mean particle size of TA-loaded NLCs of 139 nm with PDI of 0.024 and a encapsulation efficiency was 86% [105]. The in vitro results showed a significant quantity of TA on the epidermis when treated with TA-loaded NLCs suspension, decreasing the occurrence of adverse effects associated with the corticoid [105].

Silva et al. [109] developed clobetasol propionate-loaded NLCs uncoated (CP-NLC) and coated with chitosan (CP-NLC-C) to efficiently deliver the drug at the epidermal layer. The lipid nanoparticles in the absence and presence of chitosan displayed diameters of 125 nm and 258 nm, PDI of 0.25 and 0.29, zeta potential of −40.0 mV and +33.9 mV, and encapsulation efficiency of 93% and 90%, respectively [109]. Both chitosan-coated and chitosan-uncoated NLCs enhanced the amount of clobetasol propionate reaching the epidermis upon topical application.

#### 2.1.3. Hybrid Nanoparticles

Lipid-polymer hybrid nanosystems were developed to overcome the limitations of polymeric and lipid nanocarriers while maintaining the advantages of these systems. So far, these systems display improved performance in terms of loading capacity, controlled drug release, biocompatibility, and stability [111]. Thus, Pukale and coauthors [110] designed monolithic lipid-polymer hybrid nanoparticles to load clobetasol propionate for the topical management of psoriasis. The physicochemical properties of the hybrid nanoparticles were promising with average size of 95 nm, PDI of 0.213, and encapsulation efficiency of 84%. In vitro and ex vivo studies also showed that these nanocarriers display sustained drug release for 7 days, enhanced cellular uptake in HaCat cells, and high skin penetration to dermis and viable epidermis of psoriatic skin from *Swiss albino* mice. Furthermore, preclinical efficacy studies on *Swiss albino* mice with psoriasis-like inflammation showed that the treatment with a gel containing the hybrid nanocarriers improved the therapeutic efficacy compared to a marketed product [110].

Overall, the development of organic nanocarriers, which display high biocompatibility and efficient glucocorticoid loading, seem to be an efficient deliver strategy to improve the drugs’ skin penetration and reduce their side effects.

### 2.2. Topical Nanodelivery of Ceramides

As described earlier, CERs account for 50% of the SC lipids and they play key roles in maintaining the cutaneous barrier function and, as such, the pathophysiology of skin disorders associated with barrier impairment is usually associated with reduced CER levels [18,112].

Since the delivery of exogenous CERs has demonstrated beneficial effects in restoring the skin barrier function, various CERs were identified and synthesized for therapeutic and cosmetic applications [45,113]. Thus, different products containing such compounds are available in the market for skin disorders therapy: EpiCeram^®^ emulsion (Promius Pharma LLC, Bridgewater, NJ, USA), which showed safety and efficacy in children and was approved by FDA in 2006 for dermatoses therapy [114]; Lipobase^®^ Repair (Astellas, Czech Republic), intended for the direct replacement of lipids in SC and indicated for therapy of AD and psoriasis; CeraVe^®,^ which is intended for the relief of dry and itchy skin [115]. In 2007, Vávrová and colleagues [116] assessed the impact of a cream containing an analogue of CER[NS], N-tetracosanoyl-(L)-serine tetradecyl ester and compared it with the Lipobase^®^ Repair formulation. The tests were performed on excised human skin, whose barrier was disturbed by solvent-induced lipid extraction, tape stripping, and surfactant treatment. The ex vivo results showed that the cream containing the CER[NS] analogue not only decreased TEWL, but also improved the skin barrier characteristics, compared to the Lipobase^®^ Repair cream and the lipid-free control cream [116]. Moreover, Bellew and del Rosso [117] showed that after 30 days of using EpiCeram^®^ in combination with 12% ammonium lactate Lac-Hydrin^®^ (Ranbaxy Laboratories, Jacksonville, FL, USA) in patients suffering from ichthyosis, there was a significant decrease in xerotic skin and ichthyotic scaling [117]. In their work, Koppes et al. [118] conducted a randomized, double-blind, emollient- and glucocorticoid-controlled trial to evaluate the effects of a cream containing CERs and magnesium (CER-Mg) in comparison with a Unguentum leniens containing hydrocortisone in 100 patients with AD. After 45 days, there were significant differences in skin hydration, NMF, and TEWL rates between groups, and CER-Mg cream provided the best results [118]. More recently, Ishida and coworkers [119] developed a topical formulation containing designed pseudoceramides for patients suffering from AD. After 1 month of treatment, both skin symptoms and TEWL decreased, in contrast to healthy volunteers. The skin CERs were identified in both groups through analytical techniques, and it was shown that the treatment with pseudoceramides contributed to change the CERs composition of skin from an atopic profile into a healthy profile [119].

As stated above, commercial products made of CERs are available for topical treatments as creams, moisturizers, and lotions. However, there is an impasse related to skin permeability and penetration of CERs through the topical via. It was reported that certain classes of CERs do not penetrate well through the SC, resulting in no notable clinical responses [120]. These divergences in skin permeation may be related to the presence of penetration enhancers in the formulations (eg. ethoxydiglycol), as well as to the skin health condition; for instance, dry skin with low levels of endogenous CERs tend to facilitate the CERs passage by the topical via [121,122].

In this sense, the inclusion of CERs in nanocarriers may be a good strategy to improve their skin permeation, thus providing the rationale for the use of nanocarriers for the delivery of CERs (Table 3).

#### 2.2.1. Polymeric Nanoparticles

Jung and collaborators [123] formulated CER-loaded chitosan-coated PLGA nanoparticles. The uncoated nanoparticles composed of PLGA and PLGA/CER had diameters of 208 nm and 227 nm, respectively. In contrast, the chitosan-coated nanoparticles displayed diameters of 214 nm and 211 nm in the absence and presence of CER, respectively. The mean zeta potential of the uncoated PLGA/CER nanoparticles was –34 mV, while the PLGA and PLGA/CER nanoparticles coated with chitosan displayed a mean zeta potential of +36 and +49 mV, respectively. This work showed that the chitosan coating improves mucoadherence and flattens the early release of CER, compared to the uncoated nanoparticles. Using a rat AD model, the authors also showed that the CER-containing polymeric nanoparticles are effective to trigger the stratum corneum regeneration, restoring its normal thickness [123].

Tessema et al. [124] developed lecithin-based microemulsions and cassava starch nanoparticles loading oat-derived CERs, which were further incorporated in a Carbopol^®^ 980 gel. The nanoparticles containing oat CERs were characterized in terms of size, PDI, and encapsulation efficiency: 162 nm, 0.171, and 85%, respectively. In vitro tests revealed that oat CERs incorporated into the nanoparticle-containing gel were able to reach the deeper layers of a multilayer membrane system and showed a prolonged release in contrast to the microemulsion-containing gel [124].

#### 2.2.2. Lipid-Based Nanoparticles

Different types of lipid-based nanoparticles were considered for the cutaneous delivery of CERs, including SLN, NLC, vesicular nanosystems, and nanoemulsions.

Gaur et al. [126] included ceramide 2 (N-stearoyl-DL-sphinganin) into SLN nanoparticles loading curcumin and the formulation containing the highest amount of CER displayed the lowest particle size (103 nm), the lowest PDI (0.187), the lowest zeta potential (−38 mV), and the highest entrapment efficiency (90%). These nanoparticles were also the best controlling the release of curcumin during 24 h and favoring the drug permeation in ex vivo human skin. Preclinical studies in Wistar albino rats showed that these CER-containing SLN significantly enhanced the bioavailability of curcumin with improved efficacy in terms of edema inhibition compared to a conventional formulation [126].

A CER-based NLC formulation was developed by Noh et al. [127] to evaluate its potential as a transdermal delivery system for the active compound ILTG (2′,-4′,-4-trihydroxychalcone). The average particle diameter of the ILTG-NLCs formulations ranged from 152 to 252 nm. The zeta potential of ILTG-NLCs ranged from −24 mV to −27 mV. The authors observed that the lower the amount of caprylic/capric triglyceride (liquid lipid), the lower the viscosity of NLCs formulations and the smaller the particle sizes. Liquid lipid content also increased the zeta potential value, and this was attributed to the increase in the number of carboxylic groups in the caprylic/capric triglyceride. The best result for the ILTG encapsulation efficiency (90%) was achieved using the NLC formulation with 70% of liquid lipid content. The ILTG-NLCs improved the skin permeation of the compound in comparison with a ILTG solution in propylene glycol [127].

A CER-based vesicular system, called Cerosomes, was designed by Abdelgawad et al. [129] to load tazarotene for the treatment of psoriasis. The inclusion of CERs in the vesicular system improved the drug entrapment, favored the control of the drug release, and enhanced the skin deposition of the drug. Tazarotene-loaded Cerosomes displayed better clinical efficacy than a topical marketed product [129], highlighting the potential of CER-containing nanocarriers to improve therapeutic outcomes.

So far, the use of nanocarriers seems to be a valuable strategy for the topical delivery of CERs, which may be highly beneficial in the management of skin disorders associated with epidermal barrier impairment.

## 3. Conclusions

In the epidermis, the SC and its lipid components are the main contributors to the cutaneous barrier function and, thus, are of capital importance for maintaining skin health. Alterations or disorders in the lipid composition of the SC can result in skin diseases due to irritation, lesions, and inflammatory processes, as evidenced in diseases such as xerosis, ichthyosis, atopic dermatitis, and psoriasis. Their current therapeutic management still has limitations and safety issues.

The encapsulation of glucocorticoids into nanodelivery systems can be foreseen as an interesting strategy that provides biocompatibility and bioavailability, allows topical administration, improving the risk-benefit ratio of glucocorticoids and eventually allowing a more effective treatment. Moreover, CERs-containing nanocarriers may provide a more efficient and sustained release of these crucial lipids to maintain and/or restore skin barrier homeostasis. Furthermore, the physicochemical properties of the nanocarriers can be modulated, allowing a specific differential therapy for each skin disease and patient.

So far, most research works on the development of glucocorticoid or CER-containing nanocarriers are focused on in vitro, ex vivo, and preclinical studies. Considering the Clinical Trials platform, only one phase 1 clinical trial was reported using uncoated paclitaxel nanoparticles to evaluate their antipsoriatic efficacy upon topical application [130]. To the best of our knowledge, to date, no clinical trials concerning glucocorticoid- or CER-containing nanocarriers already started in the scope of skin disorders with barrier impairment. To ascertain the important benefits of these nano-strategies, more clinical and toxicological studies are needed to ultimately include these nanotechnological-based therapeutic strategies in the management of diseases associated with skin barrier impairment.

## Figures and Tables

**Table 1:** Advantages and disadvantages of each type of nanocarriers and its main potential for topical skin applications.

Type of Nanocarrier		Advantages	Disadvantages	Potential for Topical Applications	Ref.
**Lipid-based nanoparticles**	- Vesicular systems	High flexibility, biocompatibility, and biodegradability.	Short shelf life; low stability; low encapsulation efficacy.	Improved the penetration of drugs through the skin.	[68,74,75,76]
	- Solid lipid nanoparticles (SLN)	High biocompatibility and biodegradability; Easier to scale-up the production.	Long-term instability in terms of size and loading capacity.	Improved skin permeability and retention time of drugs in SC; Decreased water loss and enhanced skin hydration.	[77,78,79]
	- Nanostructured lipid carriers (NLC)	Greater stability and higher loading capacity than SLN; Reduction of the drug expulsion during storage.	Long-term instability is still possible.		[76,77,79,80]
**Polymeric nanoparticles**	- Natural	Highly biocompatible; Non-toxic.	Susceptibility to pH variations; Low reproducibility; Prone to degradation; Potentially antigenic.	Decreased adverse reactions due to applied drugs; decreased skin rash; enhanced skin permeation; increased retention of lipophilic drugs.	[68,78,81,82]
	- Synthetic	Higher stability in biological fluids and controllable physicochemical properties; High versatility and ease of production; Low costs.	Some polymers’ cytotoxicity.		[78,82,83,84]
**Metallic nanoparticles**	- Gold nanoparticles (AuNPs)	Easily prepared, functionalized and dispersed in liquids; Versatile platform for therapeutic agents.	Biosafety issues of gold; high costs.	Antioxidant and antimicrobial activity; antiaging properties.	[85,86,87]
	- Silver nanoparticles (AgNPs)	Application as antimicrobial, anti-inflammatory, antiangiogenic, and anticancer agent; green chemistry techniques show high yield, solubility and high stability.	Conventional methods of preparation are considered expensive and use toxic substances; biosafety issues of silver.	Antimicrobial activity potential for wound- or burn-dressings.	[88,89]
**Silica nanoparticles**		Good biocompatibility and controllable size; easy surface and pore functionalization; high drug loading; good thermal and chemical stability.	Difficult production protocols; instability of the colloidal suspensions.	Possibility to load hydrophylic/lipophilic compounds.	[68,90]

**Table 2:** Summary of glucocorticoid-loaded nanodelivery systems designed for topical applications.

Type of Nanoparticle	Composition	Glucocorticoid	Type of study	Ref.
Polymeric nanoparticles	Ethyl cellulose and Eudragit^®^	Dexamethasone	In vitro & Ex vivo	[98]
	Eudragit^®^ L100	Dexamethasone	In vitro & Ex vivo	[99]
	Poly (ε-caprolactone)	Hydrocortisone	In vitro	[100]
	Chitosan	Hydrocortisone	In vitro & Preclinical (mice)	[101]
	Eudragit^®^ RS 100	Dexamethasone	In vitro & Ex vivo	[102]
	Hyaluronic acid-coated chitosan	Betamethasone	In vitro & Ex vivo	[103]
Solid lipid nanoparticles	Compritol^®^ 888 ATO, Poloxamer^®^ 188	Prednisolone, diester prednicarbate, betamethasone 17-valerate	In vitro & Ex vivo	[104]
	Compritol^®^ 888 ATO, Poloxamer^®^ 188, soya lecithin	Triamcinolone Acetonide	In vitro & Ex vivo	[105]
	Glycerol monostearate	Halobetasol Propionate	Ex vivo & Preclinical (rabbit)	[106]
Nanostructured lipid carriers	Compritol^®^ 888 ATO and Miglyol^®^ 812	Fluocinolone Acetonide	In vitro & Ex vivo	[107]
	Compritol^®^ 888 ATO and Miglyol^®^ 812	Triamcinolone Acetonide	In vitro & Ex vivo	[108]
	Stearic acid, oleic acid, and lecithin. Chitosan for coating.	Clobetasol Propionate	In vitro & Ex vivo	[109]
Hybrid nanoparticles	mPEG-PLA copolymer, Precirol^®^ ATO5 and glycerol monostearate, linoleic and oleic acid	Clobetasol Propionate	In vitro, Ex vivo & Preclinical (mice)	[110]

**Table 3:** Summary of ceramide-loaded nanodelivery systems designed for topical applications.

Type of Nanoparticle	Composition	Ceramides	Type of Study	Ref.
Polymeric nanoparticles	Chitosan-coated PLGA	NR	In vitro, Ex vivo & Preclinical (rats)	[123]
	Cassava starch acetate	Oat glucosylceramides	In vitro & Ex vivo	[124]
Solid lipid nanoparticles	Lecithin and caprylic/capric triglycerides	Egg-ceramides	In vitro	[125]
	Glyceryl monostearate, stearic acid, and palmitic acid	N-stearoyl-DL-sphinganin	In vito, Ex vivo & Preclinical (rats)	[126]
Nanostructured lipid carriers	Cholesterol and caprylic-capric triglyceride	DS-Ceramide Y30	In vitro & Ex vivo	[127]
Nanoemulsions	Lipoid E-80^®^, cholesterol, palmitic acid and α-tocopherol	Ceramide III, ceramide IIIB, and phytosphingosine (PS)	In vitro	[128]
Vesicular nanosystems	Epikuron 200, Sodium deoxycholate and tween 80	Ceramide VI	In vitro, Ex vivo & Clinical	[129]

NR: Not referred; PLGA: Polylactic-co-glycolic acid.
